# Supplementary material for: In Vitro Evaluation of Antimicrobial Effect of Phytobiotics Mixture on Salmonella spp. Isolated from Chicken Broiler
Source: Antibiotics (Basel). 2022 Jun 28;11(7):868. doi: 10.3390/antibiotics11070868 (PMC9312233; doi:10.3390/antibiotics11070868)
Supplement: Supplementary file 1 [file antibiotics-11-00868-s001.zip › antibiotics-1783214-supplementary.pdf]

In vitro evaluation of antimicrobial effect of phytobiotics mixture on *Salmonella* spp. isolated from chicken broiler.

Hubert Iwiński[1,5*], Karolina Wódz[2], Karolina Chodkowska[3,5], Tomasz Nowak[2], Henryk Różański [4,5],

[1] Department of Chemistry, The Faculty of Food Science, Wrocław University Of Environmental And Life Sciences, C.K. Norwida 25,50-375 Wrocław, Poland; hubert.iwinski@upwr.edu.pl (H.I.)
[2] Laboratory of Molecular Biology, Vet-Lab Brudzew, Turkowska 58c, 62-720 Brudzew, Poland; karolina.wodz@labbrudzew.pl (K.W.); tomasz@labbrudzew.pl (T.N.)
[3] Krzyżanowski Partners Spółka z o.o., Zakładowa 7,26-670 Pionki, Poland; k.chodkowska@jkrzyzanowski.pl (K.Ch.)
[4] Laboratory of Industrial and Experimental Biology, Institute for Health and Economics, Carpathian State College in Krosno, Rynek 1, 38-400 Krosno, Poland; rozanski@rozanski.ch (H.R.)
[5] AdiFeed Sp. z o.o., Opaczewska 02-201 Warszawa
* Correspondence: hubert.iwinski@upwr.edu.pl

Table of content

Table S1. A summary of the results of biochemical reactions for the strain of *Salmonella* isolated from different samples. 1

Figure S1. MIC evaluation of *S*. Typhimurium 3

Figure S2. MIC evaluation of *S*. Enteritidis 4

Figure S3. MIC evaluation of *S*.Kentucky 4

Figure S4. Negative control 5

Table S1. A summary of the results of biochemical reactions for the strain of *Salmonella* isolated from different samples.

**REACTIONS/ENZYMES**	**VITEK2 *Salmonella enterica* ssp. *enterica Salmonella* Typhimurium 1/2/3/4**	**VITEK2 *Salmonella enterica* ssp. *enterica Salmonella* Kentucky 1/2**	**VITEK2 *Salmonella enterica* ssp. *enterica Salmonella* Enteritidis 1/2/3/4/5/6**
APPA	-/-/-/-	-/-	-/-/-/-/-/-
ADO	-/-/-/-	-/-	-/-/-/-/-/-
PyrA	+/-/-/-	-/-	-/-/-/-/+/-
IARL	-/-/-/-	-/-	-/-/-/-/-/-

dCEL	-/-/-/-	-/-	-/-/-/-/-/-
BGAL	+/-/-/-	+/+	-/-/-/-/-/+
H2S	+/+/+/+	+/+	+/+/+/+/+/+
BNAG	-/-/-/-	-/-	-/-/-/-/-/-
AGLTp	-/-/-/-	-/-	-/-/-/-/-/-
dGLU	+/+/+/+	+/+	+/+/+/+/+/+
GGT	+/+/-/-	-/	+/+/-/-/-/+
OFF	+/+/+/+	+/+	+/+/+/+/+/+
BGLU	-/-/-/-	-/-	-/-/-/-/-/-
dMAL	+/+/+/+	+/+	+/+/+/+/+/+
dMAN	+/+/+/+	+/+	+/+/+/+/+/+
dMNE	+/+/+/+	+/+	+/+/+/+/+/+
BXYL	-/-/-/-	-/-	-/-/-/-/-/-
BAlap	-/-/-/-	-/-	-/-/-/-/-/-
ProA	+/-/-/-	-/-	+/-/-/-/-/-
LIP	-/-/-/-	-/-	-/-/-/-/-/-
PLE	-/+/-/-	-/-	-/-/-/-/-/-
TyrA	+/+/+/-	-/-	-/+/+/-/-/-
URE	-/-/-/-	-/-	-/-/-/-/-/-
dSOR	+/+/+/+	+/+	+/+/+/+/+/+
SAC	-/-/-/-	-/-	-/-/-/-/-/-
dTAG	+/+/+/+	-/-	+/+/+/+/+/-
dTRE	+/+/+/+	+/+	+/+/+/+/+/+
CIT	+/+/+/+	-/+	+/+/+/+/+/+
MNT	-/-/-/-	-/+	-/+/-/-/-/-
5KG	-/-	-/-	-/-/-/-/-/-
lLATk	+/-/-/-	-/-	-/-/-/-/-/-
AGLU	-/-/-/-	-/-	-/-/-/-/-/-
SUCT	+/-/-/-	-/-	-/-/-/-/-/+
NAGA	-/-/-/-	-/-	-/-/-/-/-/-
AGAL	+/+/+/+	+/+	+/+/+/+/+/+
PHOS	-/+/+/+/	-/-	-/-/+/-/-/-
GlyA	-/-/-/-	-/-	-/-/-/-/-/-
ODC	+/+/+/+	+/+	+/+/+/+/+/+
LDC	+/+/+/+	+/+	+/+/+/+/+/+
lHISa	-/-/-/-	-/-	-/-/-/-/-/-
CMT	+/+/+/+	+/+	+/+/+/+/+/+
BGUR	-/-/-/-	-/-	-/-/-/-/-/-
O129R	+/-/+/-	+/-	+/+/-/-/-/-
GGAA	-/-/-/-	-/-	-/-/-/-/-/-
lMLTa	-/-/-/-	-/-	-/-/-/-/-/-
ELLM	-/-/-/-	-/-	-/-/-/-/-/-
lLATa	-/-/-/-	-/-	-/-/-/-/-/-

Common reaction for VITEK, API 20 E and Lab-made tests: BGAL/ONPG - beta-galactosidase, H2S - H2S production, dGLU/GLU - D-glucose, dMAN/MAN - D-mannitol, URE – urease, dSOR/SOR - D-sorbitol (not

includet Lab-made), SAC - saccharose/sucrose, CIT - citrate (sodium), ODC - ornithine decarboxylase (not includet Lab-made), LDC - lysine decarboxylase, **VITEK:** APPA - Ala-Phe-Pro-arylamidase, ADO – adonitol, PyrA - L-pyrrolydonyl-arylamidase, IARL - L-arabitol, dCEL - D-cellobiose, BNAG - beta-n-acetyl-glucosaminidase, AGLTp - Glutamyl Arylamidase pNA, GGT - gamma-glutamyl-transferase, OFF - fermentation/ glucose, BGLU - beta-glucosidase, dMAL - D-maltose, dMNE - D-mannose, BXYL - beta-xylosidase, BAlap - beta-alanine arylamidase pNA, PrpA - L-Proline arylamidase, LIP – lipase, PLE – palatinose, TyrA - Tyrosine arylamidase, dTAG - D-tagatose, dTRE - D-trehalose, MNT – malonate, 5KG - 5-keto-d-gluconate, lLATk - L-lactate alkalinisation, AGLU - alpha-glucosidase, SUCT - succinate alkalinisation, NAGA - beta-N-acetyl-galactosaminidase, AGAL - alpha-galactosidase, PHOS – phosphatase, GlyA - glycine arylamidase, lHISa - L-histidine assimilation, CMT – coumarate, BGUR - beta-glucuronidase, O129R - O/129 resistance (comp.vibrio.), GGAA - Glu-Gly-Arg-arylamidase, lMLTa - L-malate assimilation, ELLM – ellman, lLATa - L-lactate assimilation **API 20E:** ADH - arginine dihydrolase, TDA - tryptophane deaminase, IND - indole production, VP - acetoin production (Voges Proskauer), GEL – gelatinase, INO – inositol, RHA – rhamnose, MEL – melibiose, AMY - amygdalin, ARA – arabinose

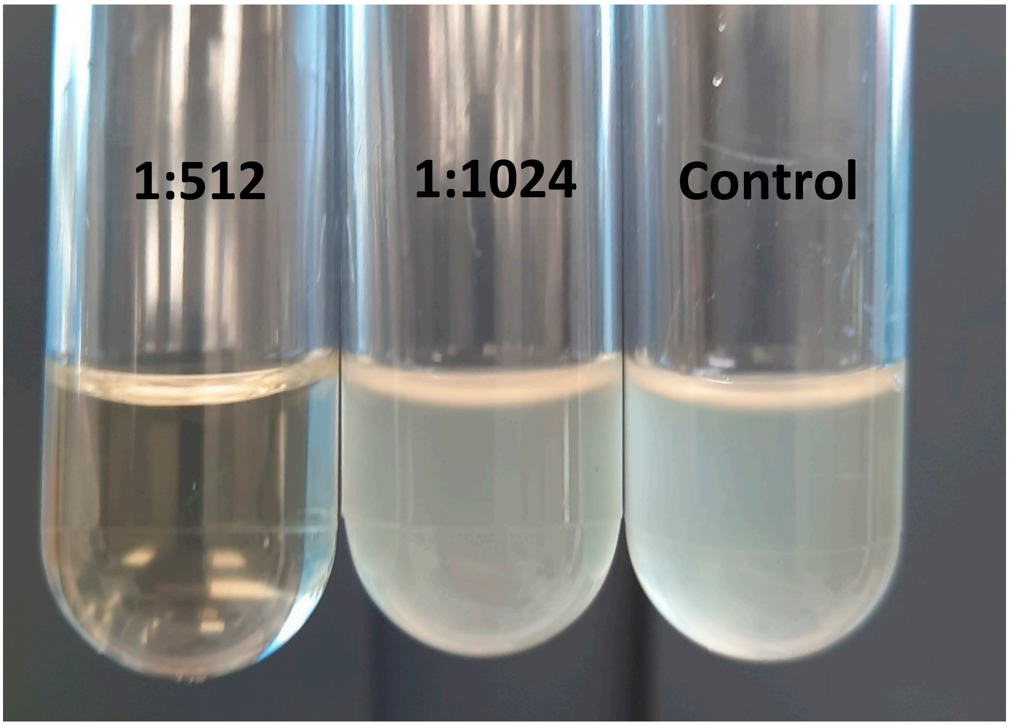

Figure S1. MIC evaluation of *S.* Typhimurium

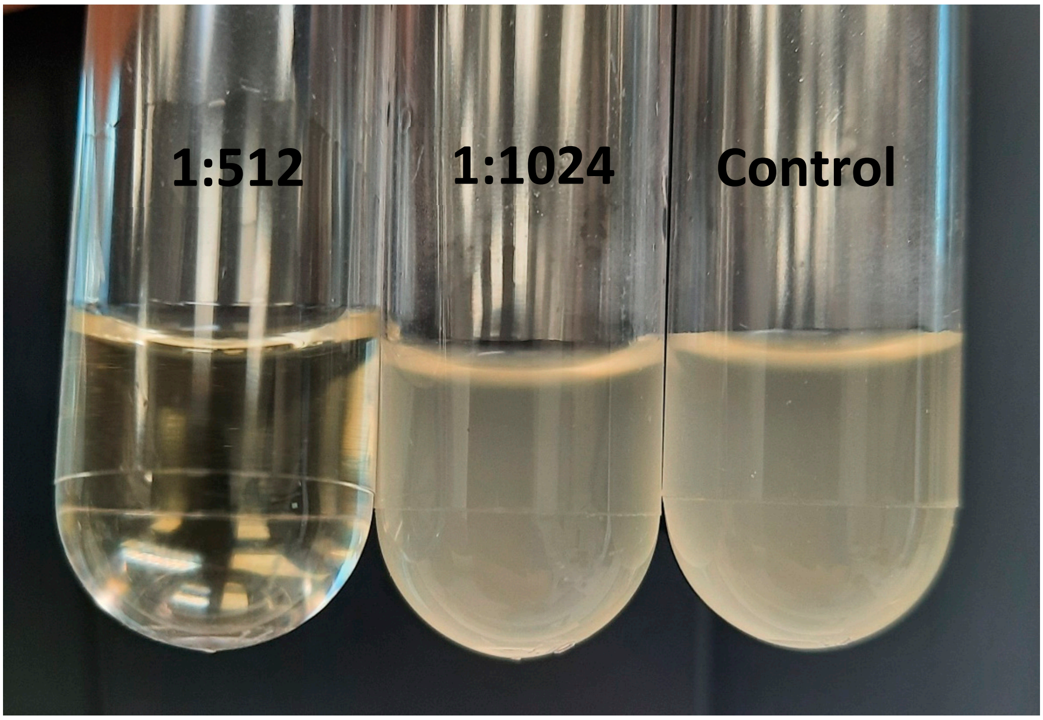

Figure S2. MIC evaluation of *S*. Enteritidis

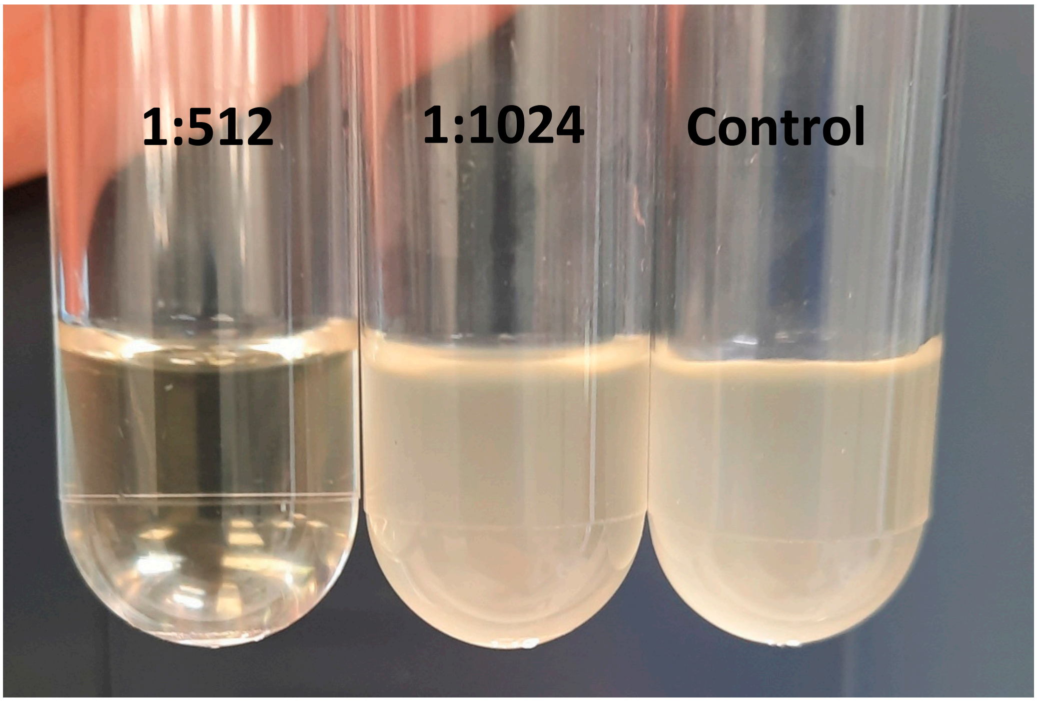

Figure S3. MIC evaluation of *S*.Kentucky

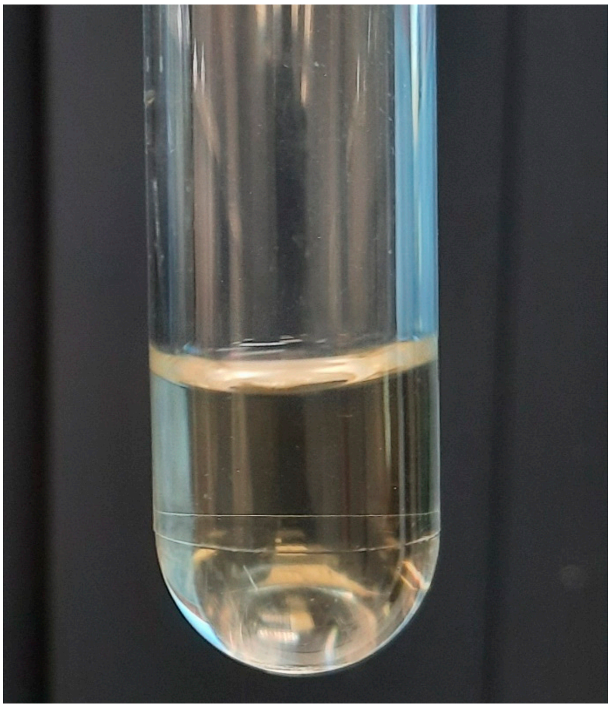

Figure S4. Negative control
